# Genotypic Differences in Triacylglycerol-Centered Lipid Metabolism at the Kernel Developing Stage in Three Representative Yunnan Macadamia Cultivars Revealed by Integrated Omics

**DOI:** 10.3390/foods15213830

**Published:** 2026-10-26

**Authors:** Yuanqi Wang, Siyuan Zhang, Yurou Wang, Mingju Hu, Hùng Tiến Lương, Tao Liu, Ran Tang, Xiaoyu Xu

**Affiliations:** 1 College of Agronomy and Biotechnology, Yunnan Agricultural University, Kunming 650201, China; 2 Faculty of Biotechnology and Food Technology, Thai Nguyen University of Agriculture and Forestry, Thai Nguyen 252160, Vietnam

**Keywords:** *Macadamia integrifolia* Maiden & Betche, triacylglycerol, lipid metabolism, developing kernel, integrated omics

## Abstract

*Macadamia integrifolia* is an economically important woody oil crop valued for its high monounsaturated fatty acid content and excellent kernel nutritional quality. Although it has great industrial potential, variety-related differences in lipid metabolism and kernel quality are still poorly understood. Resolving these differences will support targeted quality improvement and molecular-breeding efforts. In this study, three representative macadamia cultivars, namely DS1, GR1 and O.C., which are widely cultivated in Yunnan province of China, were employed to investigate the dynamic variations in kernel quality traits across three physiological stages. Integrated transcriptomic, metabolomic, and lipidomic analyses were applied at the kernel development stage to characterize the metabolic differentiation. Multi-omics results revealed distinct carbon allocation and lipid accumulation patterns observed among the three cultivars. Variety-related differences in oil yield and nutrient profiles were associated with altered gene expressions and metabolite levels linked to fatty acid biosynthesis, triacylglycerol (TG) assembly and oil-body formation. Differences in lipid and carbon metabolite accumulation also contributed to these phenotypic variations. This study preliminarily identified candidate genes and pathways associated with TG-centered lipid metabolism in developing macadamia kernels at a critical developmental stage, based on samples collected from single trees per cultivar.

## 1. Introduction

Macadamia (*Macadamia integrifolia* Maiden & Betche) is an important woody oil crop worldwide, rich in various nutrients, and is known as the ‘King of Nuts’ [1]. Yunnan Province of China has now become the core region for global macadamia production, processing, and consumption, accounting for more than half of the total global planting area [2]. *M. integrifolia* in Yunnan was mainly introduced from Australia, and after years of continuous gene exchange and ecological adaptation, it has formed a rich germplasm resource population, showing broad industrial prospects [3,4,5].

The edible part of macadamia is the kernel, in which lipids are the main component, accounting for more than 60%, mainly in the form of storage lipid triacylglycerol (TG) [6]. Macadamia TG contains 15 fatty acids, among which the monounsaturated fatty acid (MUFA) oleic acid (C18:1) has the highest proportion (>60%), followed by palmitoleic acid (C16:1), with a content between 20% and 30% [7,8]. Oleic acid has various excellent properties such as resistance to high temperatures, long-term storage stability, and antioxidant activity, and exhibits significant effects in food processing and storage, human health care, and the treatment and prevention of chronic cardiovascular and cerebrovascular diseases [9,10,11]. Palmitoleic acid has favorable application prospects in weight loss, anti-inflammation, treatment of metabolic syndrome, and the development of high-end cosmetics [12,13]. Therefore, macadamia has been widely popular as a raw material for healthy foods. However, current research on macadamia nuts is still largely concentrated in areas such as food processing [10], identification of active components [14,15], improvement of cultivation practices [16,17], and pest and disease control [18], while reports on the regulation of lipid metabolism remain relatively limited, which has restricted the improvement of macadamia nut quality and breeding innovation.

Plant lipid metabolism is a highly regulated and complex process [19,20]. Despite the largely conserved and well-characterized plant lipid metabolic pathways [21,22], differences in functional-gene expression, genetic background, growth conditions, and enzyme substrate preferences could produce large variations in lipid classes, lipid contents, and final fatty-acid profiles [23,24,25]. Shi et al. explored the differences in lipid metabolism among three important physiological stages during the maturation of macadamia nut kernels using a combined transcriptomic and lipidomic analysis approach [26]. The results showed that differential expression of functional genes such as glycerol-3-phosphate (G3P) acyltransferase (GPAT), diacylglycerol (DG) kinase, pyruvate kinase 1, phospholipase C (PLC), 2,3-ketoacyl-acyl carrier protein reductase, and linoleate 9S-lipoxygenase, together with differential accumulation of important storage and functional lipid components such as TG, DG, phosphatidylethanolamine (PE), and phosphatidylcholine (PC), were important factors causing changes in fat synthesis, storage, and quality in macadamia nuts. However, due to insufficient genome information, more information could not be effectively annotated at that time. Hu et al. analyzed fatty acid differences among 197 macadamia germplasm resources and discovered a novel desaturation mechanism regulating the two major saturated fatty acids (SFAs), palmitic acid (C16:0) and stearic acid (C18:0). It was found that some materials exhibited a preference for stearic acid synthesis, and the mechanism also had a considerable effect on the biosynthesis of palmitoleic acid and oleic acid, reflecting a pronounced differentiation in lipid metabolism regulation among macadamia nuts derived from different genetic origins [5,27].

Benefiting from the rapid development of modern genomic sequencing technology, the whole-genome information of macadamia has now been revealed, providing an important basis for deeply understanding the molecular genetic mechanisms of macadamia. Using the ‘Kau’ cultivar as the research subject, Lin et al. constructed a chromosome-level whole-genome map of macadamia using third-generation sequencing technology for the first time, finding that its genome size is 794 Mb, and through de novo annotation and homologous protein alignment, a total of 37,728 protein-coding sequences were predicted [28]. Genetic analysis revealed that the related functional genes were significantly enriched in pathways such as fatty acid biosynthesis, seed coat development, and heat stress response, which is generally consistent with the genome sequencing results of ‘GR1’, a cultivar widely planted in China [29]. Additionally, further bioinformatics analysis also found that macadamia possesses 10,853 unique singleton proteins. With these genomic resources available, it is now feasible to further explore the lipid metabolism differences across macadamia germplasms and identify molecular factors shaping kernel quality. Such work can support cultivar breeding, quality improvement, and industrial application, and also accelerate molecular-breeding research for tropical woody oil crops [30]. The application of modern omics analysis technologies will be able to play an important role in this regard [6,28,31].

The cultivar ‘DS1’ (DS1), independently bred by Yunnan Discovery Group Macadamia Co., Ltd., together with ‘GR1’ and ‘O.C.’, two cultivars that have been promoted and cultivated in China for many years, have currently become important resources for the development of the macadamia industry in the dry-hot valley regions of Lincang and Dehong, Yunnan, exhibiting multiple advantages such as a long peak fruiting period, high yield and high oil content, strong disease resistance, and strong ecological adaptability [28,29]. This study therefore used these three representative macadamia cultivars from these regions as experimental materials and analyzed their major quality component changes across three important kernel growth and developmental stages: the fruit enlargement stage (S1), the fruit nutrient accumulation and conversion stage (S2), and the fruit ripening stage (S3). Based on phenotypic changes across S1–S3, S2 was identified as an important metabolic inflection point of kernel development. Therefore, integrated transcriptomic, metabolomic and lipidomic analyses were specifically conducted for the S2 stage to preliminarily characterize genotypic differences in gene expression and carbon-related metabolic profiles among three cultivars at this critical time point. By focusing on molecular patterns associated with the core storage lipid TG [28,31], the biosynthesis of important carbon assimilation products in the developing kernel was clarified and compared, and a set of key functional genes, metabolic pathways, and metabolites affecting macadamia quality formation were effectively identified therefrom. Our results may provide important clues for gaining deep insights into the lipid metabolism regulatory mechanisms in macadamia.

## 2. Materials and Methods

### 2.1. Experimental Materials

Three representative macadamia cultivars, DS1, GR1 (GR), and O.C. (OC), were used in this study. All trees were cultivated in Yingjiang County, Dehong Prefecture, Yunnan Province (growing altitude 825 m, 24.7° N, 97.9° E), and kernel samples were provided by Yunnan Discovery Group Macadamia Co., Ltd. Kernel samples were collected at three developmental stages: S1 stage (30–50 days after fruit set), S2 stage (60–80 days after fruit set), and S3 stage (90–110 days after fruit set). Samples were snap-frozen in liquid nitrogen and stored at −80 °C, with three biological replicates set for each stage. For each cultivar, three biological replicates corresponded to three independent healthy nuts collected from a single individual tree. Samples at different developmental stages were repeatedly harvested from the identical tree. Note: Biological replicates in this study were sampled from a single tree for each cultivar; therefore, biological variation among independent trees of the same cultivar was not assessed in the present experimental design. Multiple kernels within each replicate were pooled and fully homogenized to prepare composite samples for phenotypic measurement, transcriptomic, lipidomic and metabolomic analyses.

### 2.2. Lipid Content and Fatty Acid Analysis

The extraction of total lipids from the nut kernels and the analysis of their fatty acid composition were performed according to the method described by Gao et al. [32]. Fifty milligrams of fresh kernel samples was weighed, and after grinding them in liquid nitrogen, they were extracted for 15 min using a vortex mixer with an analytical-grade methanol–chloroform system (1:2, *v*/*v*). A volume of 1/4 of 0.75% NaCl solution was added; after thorough vortexing, the mixture was allowed to stand for 5 min, then centrifuged at 3000 rpm for 10 min. The chloroform phase was collected, dried under N_2_ to obtain the total lipid extract, and dissolved in 150 μL of chloroform. From each sample, 5 μL of the total lipid extract was taken; heptadecanoic acid (C17:0) was used as the fatty acid internal standard, and a composite standard containing fatty acids with chain lengths ranging from C4 to C25 (Sigma-Aldrich, Darmstadt, German) was used as the external standard. The samples were added to a methanol solution containing 1% sulfuric acid, mixed thoroughly, and methylated at 85 °C for 2.5 h. The resulting fatty acid methyl esters (FAMEs) were extracted with n-hexane, and the composition and content of fatty acids were determined using a gas chromatograph (Agilent 7820, Agilent Technologies, Santa Clara, CA, USA) equipped with a CP-Sil 88 gas chromatography column (100 m × 0.25 mm × 0.25 µm). MassHunter GC/MS Acquisition and Quant-My-Way software (Version 13.0, Agilent Technologies, Santa Clara, CA, USA) were used to calibrate and perform qualitative and quantitative analysis of the fatty acid peak area data. The analytical performance of this GC-FAME method was fully validated. The limit of detection (LOD) ranged from 0.2 μg/mL to 0.5 μg/mL, and the limit of quantification (LOQ) ranged from 0.5 μg/mL to 1 μg/mL for individual fatty-acid standards. Intra-day precision, expressed as relative standard deviation (RSD), was ≤2.97%, and inter-day precision (RSD) was ≤5.28%; spike recoveries for each fatty-acid standard fell within 65.8–121.0%. The calibration curves were constructed across the linear range of 1.315–105.2 μg/mL using seven gradient calibration points. Full method-validation parameters for each fatty-acid compound are provided in Supplementary Table S1. It should be emphasized that values presented in main-text Table 1 correspond to relative peak-area percentages of total fatty-acid methyl esters, not absolute calibrated concentrations. Notably, this GC-FAME assay was performed on unfractionated total lipid extracts without preliminary separation of individual lipid classes such as TG, DG and phospholipids. Accordingly, the measured fatty-acid profiles reflect the fatty-acid composition of the whole total-lipid pool and do not specifically represent TG-only fatty-acid composition.

### 2.3. Analysis of Major Carbon Assimilate Contents

#### 2.3.1. Total Starch Content

Weigh 50 mg of dried nut kernel sample, grind, and remove the sugars using 80% ethanol. Then determine the absorbance at 510 nm using the amyloglucosidase/peroxidase–glucose oxidase (GOPOD) method and calculate the starch content with a glucose standard solution [33].

#### 2.3.2. Total Protein Content

Weigh 0.2 g of fresh nut kernel sample and digest it with H_2_SO_4_-H_2_O_2_ at 380 °C until the solution becomes clear and colorless. Determine the nitrogen content using a Kjeldahl nitrogen analyzer (SKD-1000, Shanghai Peiou Analytical Instruments Co., Ltd., Shanghai, China) and calculate the protein content using a protein conversion factor of 6.25 [34].

#### 2.3.3. Soluble Sugar Content

Weigh 0.1 g of freeze-dried nut kernel sample. After ultrasonic extraction with deionized water, measure the absorbance at 490 nm using the phenol–sulfuric acid method and calculate the total soluble sugar content based on a glucose standard curve [35].

#### 2.3.4. Total Fiber Content

Weigh 0.5 g of the dried nut kernel sample and place it in a filter bag. Digest the sample by boiling it successively in 100 mL of 1.25% H_2_SO_4_ and 1.25% KOH. Wash the residue successively with hot water, ethanol, and ether, then dry it at 105 °C until constant weight. Incinerate the residue at 550 °C and calculate the total fiber content based on the difference in mass before and after incineration [36].

### 2.4. Comparative Omics Analysis

All omics analyses in this study were commissioned to Jiangsu Sanshu Biotechnology Co., Ltd. Prior to instrumental analysis, a 5 μL aliquot of extract from each sample was pooled in equal volumes to prepare a quality control (QC) sample for monitoring instrument stability.

#### 2.4.1. Transcriptomic Analysis

Two hundred milligrams of fresh nut kernel samples was taken, and total RNA was extracted using the CTAB (cetyltrimethylammonium bromide) method [37]. Purity and integrity of the RNA were assessed using an ultramicrovolume spectrophotometer (NanoDrop 2000, Thermo Fisher Scientific, Waltham, MA, USA) and agarose gel electrophoresis, respectively, to ensure that the OD260/280 ratio was between 1.8 and 2.0. Subsequently, total RNA meeting quality standards was used to construct cDNA libraries using an mRNA library construction kit (Thermo Fisher Scientific, Waltham, MA, USA). After verifying the insert size and quantifying the effective concentration (>2 nM), the library was subjected to paired-end sequencing on an Illumina high-throughput sequencing platform. Quality control was performed on the raw sequencing data; adapter sequences were filtered out, and reads containing excessive N bases or of low quality were removed to obtain clean reads. Clean reads were then aligned against the macadamia reference genome (*M. integrifolia* Mi.v1, https://ngdc.cncb.ac.cn/gwh/Assembly/23196/show, accessed on 12 March 2025) for validation. Based on this, gene function annotation was further performed using the *Arabidopsis thaliana* (L.) Heynh. genome (TAIR, https://www.arabidopsis.org/, accessed on 12 March 2025). Differential expression analysis was performed using DESeq2 (v1.42.0). The Wald test was adopted, and the Benjamini–Hochberg (BH) method was used for multiple-test correction to generate adjusted *p*-values (FDR). No manual pre-filtering with an FPKM threshold (such as FPKM > 1) was applied before DEG analysis; DESeq2’s built-in independent filtering based on normalized mean counts was automatically implemented. The selected differentially expressed genes (DEGs) were subjected to GO (Gene Ontology) functional annotation and KEGG (Kyoto Encyclopedia of Genes and Genomes) pathway enrichment analysis to identify the biological processes and metabolic pathways in which they were involved.

#### 2.4.2. Non-Targeted Metabolomics Analysis

One hundred milligrams 100 mg of freeze-dried nut sample was weighed, thawed at 4 °C, and ground; 1 mL of extraction solution was then added (water:acetonitrile:isopropanol, 1:1:1, *v*/*v*). It was vortexed thoroughly for 60 s, then low-temperature ultrasonic extraction was performed at 4 °C for 30 min. Subsequently, the sample was centrifuged at 12,000 rpm under 4 °C for 10 min; the supernatant was collected and allowed to stand at −20 °C for 1 h to precipitate proteins. It was then centrifuged again at 12,000× *g* and 4 °C for 10 min; the supernatant was collected and vacuum-dried before resuspending in 200 μL of 30% acetonitrile with quick vortexing. Then, it was centrifuged at 14,000× *g* for 15 min; the supernatant was aspirated and prepared for mass spectrometry analysis. A Vanquish ultra-high-performance liquid chromatography (UHPLC) system coupled with a Q Exactive HFX high-resolution mass spectrometer (Thermo Fisher Scientific, Waltham, MA, USA) was used, with a Waters HSS T3 column (100 × 2.1 mm, 1.8 μm). Mobile phase A consisted of a 0.1% formic acid aqueous solution, and mobile phase B consisted of a 0.1% formic acid–acetonitrile solution. The flow rate was 0.3 mL/min, the column temperature was 40 °C, and the injection volume was 2 μL. The gradient elution program was set according to the protocol described by Ren et al. [38]. Mass spectrometry was performed using an ESI source in simultaneous positive and negative ion Full MS-ddMS^2^ scan mode, with a first-stage scan range of 70–1050 Da, a resolution of 70,000, and a second-stage resolution of 17,500. Raw data were processed using Progenesis QI software (Version 2.4, Waters Corporation, Milford, MA, USA) for baseline filtering, peak identification, integration, retention time correction, and peak alignment, yielding a data matrix containing retention time, mass-to-charge ratio, and peak intensity. Metabolite identification was performed by comparing experimental precursor exact mass (mass deviation < 10 ppm) and MS/MS fragmentation spectra against Sanshu company’s in-house database and public databases including HMDB (http://www.hmdb.ca/, accessed on 12 March 2025) and METLIN (https://metlin.scripps.edu/, accessed on 12 March 2025). The MS-MS match similarity threshold was set at ≥70%. All metabolite annotations in this work correspond to MSI Level 2 (putatively annotated compounds), according to the Metabolomics Standards Initiative classification, supported by accurate-mass matching, adduct assignment and MS-MS spectral similarity, without verification against authentic chemical standards analyzed under identical LC-MS conditions. Precursor experimental *m*/*z*, theoretical *m*/*z*, mass error, retention time, and assigned adduct for each annotated metabolite are summarized in Supplementary Table S2. Complete lists of major diagnostic MS/MS fragment ions were not available for all features in the current dataset. Data filtering criteria: Only variables with non-zero values accounting for more than 80% of the data in any given sample group were retained, and variables with a relative standard deviation (RSD) of ≥30% in QC samples were excluded.

#### 2.4.3. Non-Targeted Lipidomic Analysis

Fifty milligrams of freeze-dried nut sample was weighed and ground, and then 400 μL of methyl tert-butyl ether and 80 μL of methanol were added. It was vortexed for 30 s and centrifuged at 3000 rpm for 15 min. The upper organic phase was collected and blow-dried with N_2_; the sample was then redissolved in 100 μL of dichloromethane–methanol (1:1, *v*/*v*) to resuspend the sample for analysis. The chromatographic column, instrument system, and conditions were consistent with those used for non-targeted metabolomics. Mobile phase A consisted of acetonitrile:water (6:4, *v*/*v*, containing 10 mM ammonium acetate), and mobile phase B consisted of acetonitrile:isopropanol (1:9, *v*/*v*, containing 10 mM ammonium acetate). Flow rate: 0.3 mL/min; gradient elution program: maintain 30% B from 0 to 4.0 min, linearly increase to 100% B from 4.0 to 22.0 min, decrease to 30% B from 22.0 to 22.1 min, maintain 30% B from 22.1 to 26.0 min. Mass spectrometry used an ESI source with separate collection of positive and negative ions. The first-stage scan range was 80–1200 Da, with a resolution of 70,000, and the second-stage resolution was 17,500, sheath gas was 60 arb, auxiliary gas was 10 arb, and the ion spray voltage was ±3000/−2800 V. Raw data were processed using LipidSearch 5.0 software (Thermo Fisher Scientific, Waltham, MA, USA) for lipid molecular identification and data preprocessing, with the following key parameters set: precursor ion mass deviation of 5 ppm, product ion mass deviation of 5 ppm, and product ion intensity threshold of 5%. Lipid identification was based on the LipidSearch software’s built-in database (covering 8 major classes, over 300 subclasses, and containing data on more than 1.5 million lipid ions and their predicted fragment ions), utilizing identification algorithms for parent ions, daughter ions, and neutral loss scans to enable systematic lipid qualitative and quantitative analysis. Mass spectrometry used an ESI source with separate acquisition for positive-ion and negative-ion modes. For positive-ion mode, spray voltage was +3000 V; for negative-ion mode, spray voltage was −2800 V. Sheath gas was set to 60 arb, auxiliary gas to 10 arb, auxiliary-gas heater temperature to 350 °C, and ion-transfer tube temperature to 320 °C. The first-stage scan range was 80–1200 Da, MS1 resolution was 70,000, and MS/MS resolution was 17,500. The lipid peak area data extracted by LipidSearch were filtered to remove samples with zero expression across all samples and retain lipid molecules with a proportion greater than 70%. Subsequently, data were normalized using the `metNor’ function from the `MetNormalizer’ package in R. The RSD values for each lipid molecule in the QC samples were calculated, and those with an RSD > 30% were excluded to obtain the final lipid expression matrix. Lipid molecules were classified according to the LIPID MAPS classification system, and their relative abundances were expressed as the relative percentage of total lipids or as total response values. Transcriptomic, metabolomic and lipidomic data acquisition, preprocessing and normalization were performed following standard multi-omics analytical workflows [39]. Key instrument parameters, software versions and filtering thresholds are supplemented in this section. Raw omics data were submitted to public databases to ensure the reproducibility of this study. It should be noted that the LipidSearch 5.0-driven annotation workflow in this study cannot distinguish *sn*-1/*sn*-2/*sn*-3 positional isomers of TG molecules. Therefore, the TG (X-Y-Z) notation used in the manuscript only describes the total fatty-acid composition of each TG molecular species and does not provide positional assignment for individual fatty-acid moieties on the glycerol backbone. This imposes limitations on further inference of the relative contributions of *sn*-3-preferring and *sn*-2-dependent transferases in the TG assembly, as well as assessments of oil nutritional absorption properties and TG physical-melting-crystallization characteristics.

#### 2.4.4. Screening of Differentially Accumulated Metabolites and Omics-Wide Integrated Analysis

The metabolomics data matrices from both experiments were imported into R software (Version 4.2.1, R Core Team, Vienna, Austria) or multivariate statistical analysis. Unsupervised principal component analysis (PCA) was used to examine the overall distribution across samples, and supervised orthogonal partial least squares discriminant analysis (OPLS-DA) was employed to identify metabolic differences between groups. The screening criteria for DEGs from transcriptomic data were defined as |log_2_(fold-change)| > 1 and FDR < 0.05. For non-targeted metabolomics and lipidomics data, differentially accumulated metabolites (DAMs) and differentially accumulated lipids (DALs) were screened with identical thresholds: VIP (Variable Importance in Projection) > 1.0, raw *p*-value < 0.05, and fold-change (FC) > 1.5 (up-accumulated) or <0.67 (down-accumulated). Note: FDR correction for multiple testing was only applied to transcriptomic DEG analysis; untargeted metabolomics and lipidomics analyses used unadjusted raw *p*-values combined with VIP and fold-change cut-offs to reduce false-positive signals. The identified differentially expressed substances were mapped to the KEGG database for pathway enrichment analysis, while lipid chain length and saturation analysis were performed based on the total number of carbon atoms and the total number of unsaturated bonds in the fatty acid chains of each lipid molecule. Subsequently, KEGG pathway co-enrichment analysis was performed on DEGs and DAMs from pairwise comparison groups of the three macadamia cultivars, and Venn diagrams were plotted to illustrate the overlap of shared and unique differentially expressed molecules across different comparison groups. Co-expression network analysis was performed on DEGs and DALs, significantly associated gene–lipid relationships were screened based on Pearson correlation coefficients (*p* < 0.05), and an interaction network was constructed using Cytoscape software 3.10.2 to identify core nodes regulating lipid metabolism.

### 2.5. qRT-PCR Expression Analysis

To validate transcriptome reliability, six key lipid-metabolism-related genes were selected for qRT-PCR analysis. Total RNA was isolated from S2 kernels. RNA purity (OD_260_/_280_: 1.8–2.1) was checked using a NanoDrop 2000 spectrophotometer (Thermo Fisher, Waltham, MA, USA). Reverse transcription was performed in a 20 μL system with 2.00 μL total RNA using the EasyScript One-Step gDNA Removal and cDNA Synthesis Kit: 25 °C 10 min, 42 °C 15 min, 85 °C 2 min for enzyme inactivation. Synthesized cDNA was diluted and used as a qPCR template.

qRT-PCR was run on a QuantStudio real-time PCR instrument using PerfectStart Green qPCR SuperMix. The 20 μL reaction contained 2.00 μL diluted cDNA, 0.5 μL each forward/reverse primer (10 μM), 10 μL SuperMix and 7 μL ddH_2_O. Three biological replicates with three technical replicates each were used. Cycling conditions: 94 °C 30 s; 40 cycles of 94 °C 5 s, 58 °C 15 s, 72 °C 10 s. Melt-curve analysis (60–95 °C, 0.15 °C·s^−1^ ramp) was performed to verify specific amplification. 18S rRNA served as the reference gene [40], whose stable expression across cultivars was confirmed by a narrow Ct-value distribution (18.4–20.3). Standard-curve assays verified all primer pairs with amplification efficiency 91.2–104.7% (R^2^ > 0.985). Relative expression was calculated by the 2^−ΔΔCt^ method. Pearson correlation was used to compare qRT-PCR results and log_2_-transformed RNA-Seq FPKM values. Primer sequences are listed: housekeeping gene *18S* (F: CTGAGAAACGGCTACCACATC, R: CGAAGAGCCCGGTATTGTTATT); *Mi*03Gene46198-DGAT (F: CGGCTTCTGGGTTGTCGT, R: TGCTACACGTCGTTGGCA), *Mi*03Gene27167-Oleosin (F: GACTGACGTTGGTGGCGA, R: AACGCACCCGAAGTCAGG), *Mi*03Gene35053-Caleosin (F: AAGGGTTCCGTGCGATCG, R: CCCATGTTTGCCCCTCTGT), *Mi*03Gene21702-LEC2 (F: TCTCGTTGCTGCCACCAG, R: GAGAATCAGCGGGCACGT), *Mi*03Gene36678-WRI1 (F: GGTTGCCAGGCACCATCA, R: CTCCAAGGCTGCAAGGTCA), *Mi*03Gene50951-SAD (F: GTTGGAGGTGGACCGCTC, R: TCAGGTGTGCTGGCATCG).

### 2.6. Statistical Analysis

Figures and tables were prepared and optimized using Office 2020 and GraphPad Prism 9. For phenotypic trait measurements, fatty-acid composition data, and qRT-PCR relative gene-expression data, one-way analysis of variance (one-way ANOVA) was used to evaluate differences across groups. Two key assumptions for one-way ANOVA were checked prior to analysis: the Shapiro–Wilk test for residual normality and Levene’s test for homogeneity of variances (homoscedasticity). Only after a significant omnibus F-test result (*p* < 0.05) from ANOVA was the least-significant-difference (LSD) post hoc test applied for pairwise multiple comparisons between experimental groups. Significance was defined at *p* < 0.05. SPSS 26.0 (IBM Corporation, Armonk, NY, USA) was used for the above univariate statistical calculations. Omics-related bioinformatic analyses were conducted using TBtools v2.210.

## 3. Results

### 3.1. Dynamic Variations of Main Kernel Quality in Three Macadamia Cultivars

**Figure 1:**
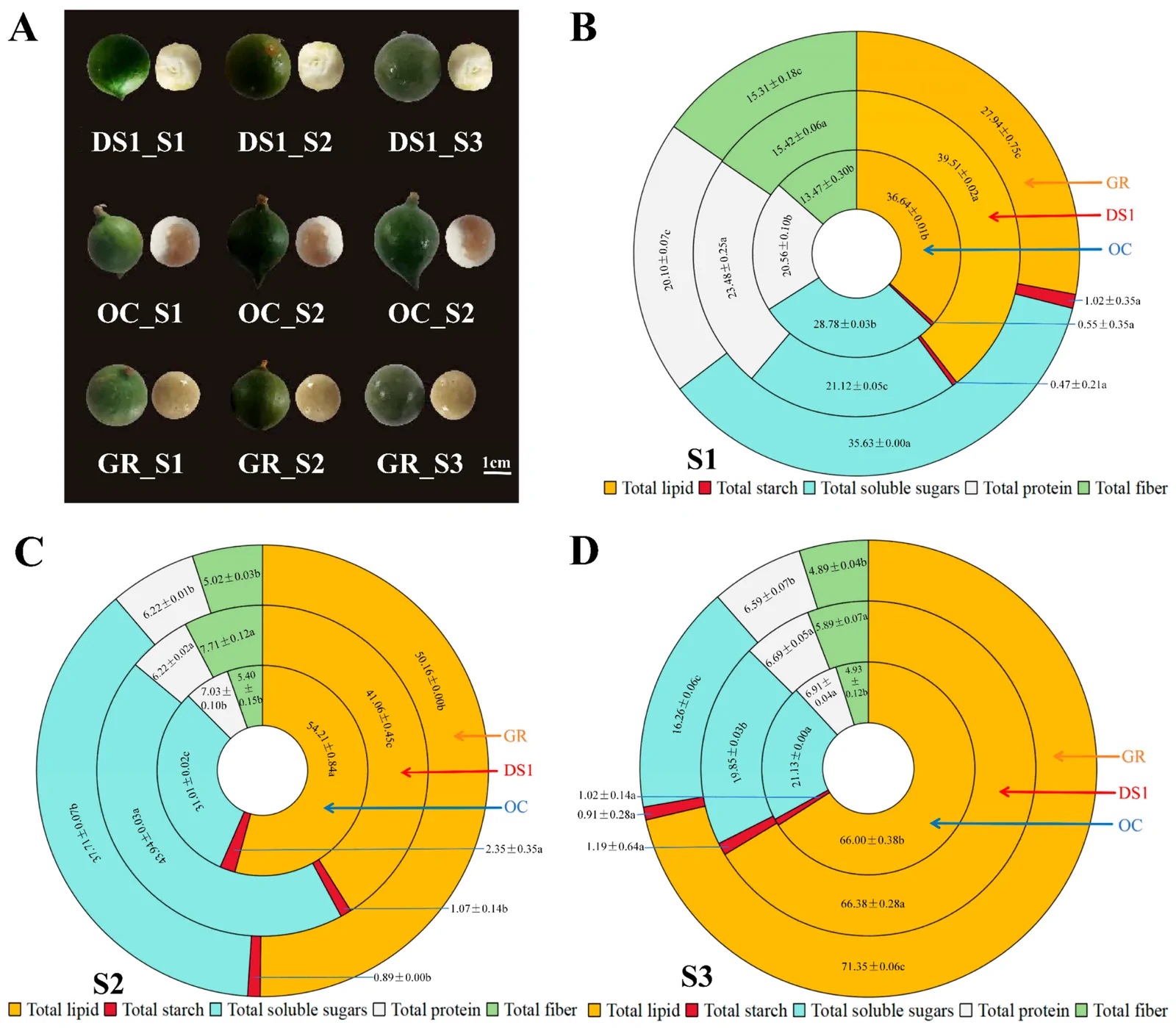
Changes in fruit and kernel morphology and major quality components (%) of three macadamia cultivars during stages S1–S3. (**A**) Fruit and kernel morphology. (**B**) Kernel quality at stage S1. (**C**) Kernel quality at stage S2. (**D**) Kernel quality at stage S3. Note: Different letters following the values indicate significant differences (*p* < 0.05) in the content of the same quality component according to the least significant difference (LSD) test.

As shown in Figure 1, among the three macadamia cultivars examined, the morphology and texture of fruits and kernels exhibited stage-specific characteristic changes during S1–S3 development (Figure 1A), with significant fluctuations in the contents of each quality component and marked differences among cultivars. All contents of lipids, proteins, soluble sugars and starch presented herein were expressed as a percentage of dry weight. At the S1 stage, soluble sugar and protein dominated kernel composition. DS1 had the highest total lipid content (39.51%), and total fiber reached 15% (Figure 1B). The S2 stage acted as a key metabolic transition point. Total lipid content rose rapidly to 41.06–54.21% across cultivars. GR showed the fastest lipid accumulation, with its lipid content nearly doubling compared with S1 and reaching 50.16%. Total soluble sugar also increased significantly across all three cultivars, with DS1 showing the most pronounced change, whereas total protein and total fiber contents both decreased several-fold compared with S1 (Figure 1C). By the S3 stage, nutrient accumulation was essentially complete, and total lipid emerged as the most predominant kernel component, with GR (71.35%) significantly higher than the other two cultivars. Concurrently, total soluble sugar, total protein, and total fiber further declined (Figure 1D), while starch content remained relatively stable but at comparatively lower levels across the developmental stages of all three cultivars. Evidently, GR exhibited stronger oil biosynthesis and metabolic activity during the middle and late stages of kernel development, whereas DS1 and OC were more active during the early developmental stage.

### 3.2. Variations in Fatty Acid Profiles of Macadamia Kernels

Table 1 presents the variations in fatty acid profile of macadamia kernels. Six medium- to long-chain fatty acids were predominantly displayed, namely palmitic acid (C16:0), palmitoleic acid (C16:1), stearic acid (C18:0), oleic acid (C18:1), and linoleic acid (C18:2), with the remainder consisting of small amounts of linolenic acid (C18:3), medium-chain fatty acids with carbon chain lengths below 16, and long-chain fatty acids (LCFAs) with carbon chain lengths above 20. Overall, the two MUFAs, palmitoleic acid and oleic acid, together accounted for over 80% of the total, although their trends during S1–S3 were opposite. Palmitoleic acid gradually decreased from approximately 30% to about 20%, whereas oleic acid increased from approximately 35% to over 60%. The proportions of palmitic acid, stearic acid, linoleic acid, and linolenic acid generally decreased significantly with fruit development, while the other fatty acids with chain lengths above C20 fluctuated only slightly. All three cultivars exhibited a consistent trend in fatty acid metabolism, in which the decline in SFAs and unsaturated fatty acids (USFAs) was accompanied by the directional accumulation of MUFAs dominated by oleic acid. However, the proportions of individual fatty acids differed markedly among cultivars and developmental stages.

In summary, analysis of the principal kernel quality traits and fatty acid profiles of the three experimental macadamia cultivars during stages S1–S3 consistently indicated that the S2 stage represented a critical turning point in quality formation, during which gene expression regulation and metabolic reallocation may have substantially influenced the quality specificity of different macadamia cultivars as well as interspecific differences. It should be noted that these GC-FAME results characterize the fatty-acid composition of the total lipid pool. At the S1 and S2 developmental stages, considerable amounts of membrane phospholipids exist in kernels, which may introduce saturated and polyunsaturated fatty acid signals and could confound the fatty acid profile originating purely from TG molecules. Multi-omics comparisons were thereafter conducted with a focus on the S2 stage to reveal the underlying molecular basis.

### 3.3. Comparative Transcriptomic Analysis Among Three Macadamia Cultivars at the S2 Stage

**Figure 2:**
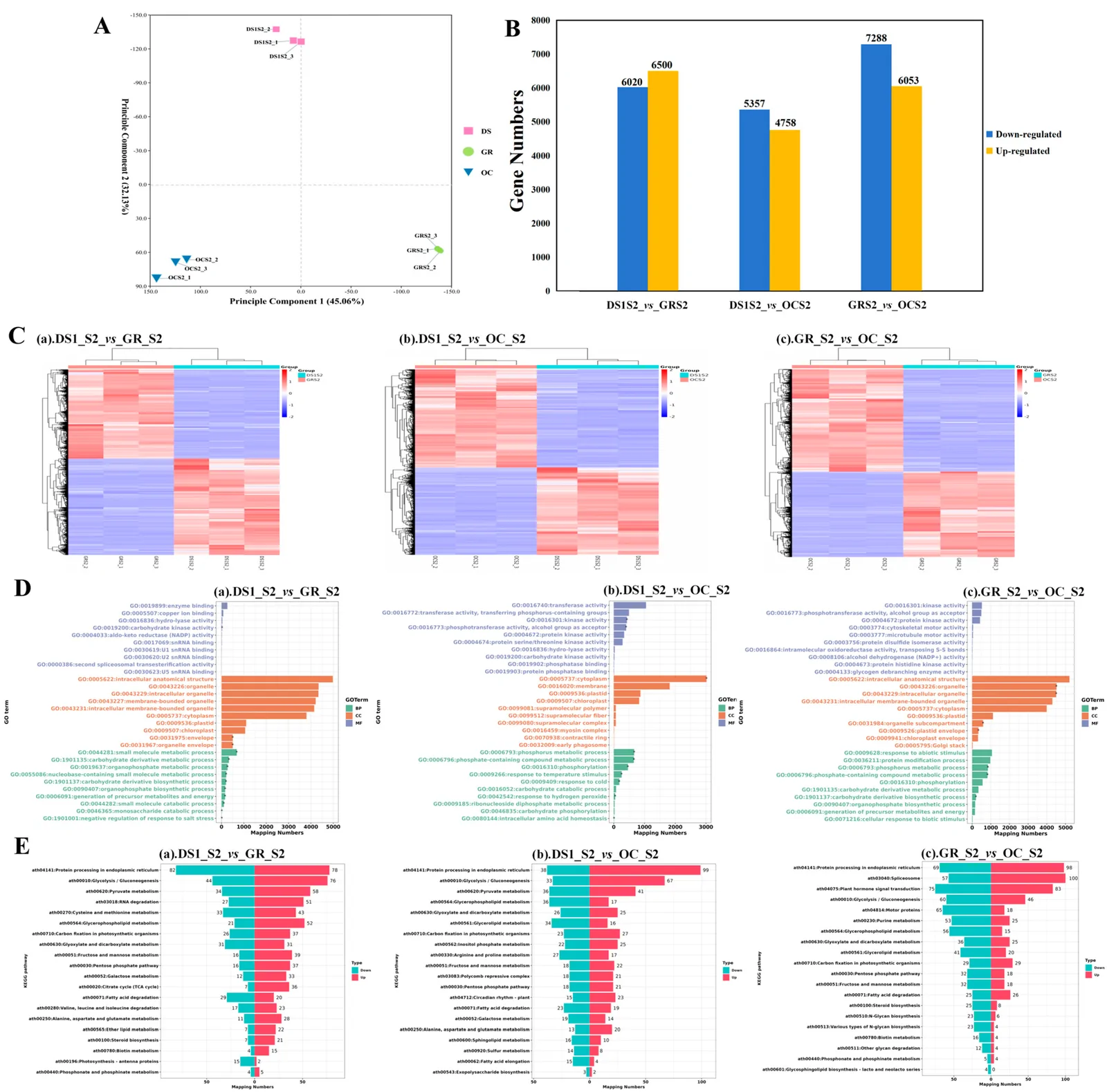
Comparative transcriptomic analysis of gene expression differences in kernels at the S2 stage among three macadamia cultivars. (**A**) PCA analysis. (**B**) Numbers of up- and down- regulated DEGs. (**C**) Heatmap visualization of DEG expression levels. (**D**) GO pathway enrichment analysis of DEGs (top 30 terms). Enrichment significance is represented by −log_10_(FDR). The significance threshold for enrichment was set at FDR < 0.05 (corresponding to −log_10_(0.05) ≈ 1.3), and only significantly enriched GO terms are displayed. The size of each dot indicates the number of genes enriched in the corresponding term. (**E**) KEGG pathway enrichment analysis of DEGs (top 20). Note: There were 3 pairwise comparison modes in total: DS1_S2 vs. GRV, DS1_S2 vs. OCV, and GR_S2 vs. OC_S2. In the DEG heatmap, red color indicates up−regulated expression, and blue color indicates down-regulated expression.

A total of 37,728 genes were detected at the S2 stage, among which 90.00%, 77.76%, and 89.19% of clean reads in DS1_S2, GR_S2, and OC_S2, respectively, could be uniquely annotated based on the whole-genome template of macadamia (Supplementary Table S3). PCA showed tight clustering of biological replicates within each cultivar. Samples from the three cultivars were clearly separated along principal component 1 (PC1, 50%), which indicates strong cultivar-specific gene-expression patterns and clear genotypic differentiation (Figure 2A). Differential expression analysis of three pairwise comparisons yielded the following DEG numbers: DS1_S2 vs. GR_S2 (12,520 DEGs, including 6500 up-regulated and 6020 down-regulated genes), DS1_S2 vs. OC_S2 (10,115 DEGs, including 4758 up-regulated and 5357 down-regulated genes), and GR_S2 vs. OC_S2 (13,341 DEGs, including 6053 up-regulated and 7288 down-regulated genes) (Figure 2B), respectively, with their FPKM values displaying a polarized heatmap distribution pattern of up-regulation and down-regulation (Figure 2C).

GO annotation across the biological process (BP), cellular component (CC), and molecular function (MF) categories indicated that differentially expressed genes at the S2 stage were predominantly concentrated in CC, with the number of enriched DEGs approximately 3–5 times higher than that in BP and MF. In the DS1 vs. GR and GR vs. OC comparison groups, most DEGs were enriched in functional modules such as intracellular anatomical structure (GO:0005622), organelle (GO:0043226), intracellular organelle (GO:0043229), and intracellular/membrane-bounded organelle (GO:0043231/0043227) (Figure 2D(a,b)), whereas DEGs in the DS1_S2 vs. OC_S2 comparison group were mainly enriched in cytoplasm (GO:0005737) and membrane (GO:0016020), but showed greater enrichment in BP and MF, such as transferase activity (GO:0016740) and phosphorus metabolic process (GO:0006793) (Figure 2D(c)). Overall, differential expression of genes related to cellular components and structures constituted the primary molecular distinction among the three macadamia cultivars, directly reflecting the influence of genetic background on the differentiation of fruit development and quality traits.

KEGG enrichment analysis revealed that protein processing in the endoplasmic reticulum (ath04141) was the metabolic pathway shared among all three comparison groups with the largest number of enriched DEGs (>130), while glycolysis/gluconeogenesis (ath00010) and pyruvate metabolism (ath00620) were relatively prominent in the DS1_S2 vs. GR_S2 and DS1_S2 vs. OC_S2 comparison groups. Although lipid metabolism pathways were not the top-ranked in terms of DEG enrichment, they exhibited significant differential divergence across the three comparison groups. For instance, glycerophospholipid metabolism (ath00563) had 73 DEGs in DS1 vs. GR (52 upregulated, 21 downregulated), 53 in DS1_S2 vs. OC_S2 (17 upregulated, 36 downregulated), and 71 in GR_S2 vs. OC_S2 (15 upregulated, 56 downregulated), and despite differences in glycerolipid metabolism (ath00561) were not reflected in DS1_S2 vs. GR_S2, more than 50 DEGs were enriched in each of the other two comparison groups (Figure 2E). Other differentially regulated metabolic pathways also involved amino acid metabolism (ath00270), phosphorus and sulfur metabolism (ath00440, ath00920), and fructose, mannose, and galactose metabolism (ath00051, ath00052), among the comparison groups, suggesting distinct metabolic discrepancies.

The comparative transcriptomic analysis results revealed that differential gene expression at the S2 stage was closely related to kernel quality formation, and GO_S2 analysis indicated that differences were concentrated in CC, suggesting cultivar-specific regulation of the synthesis, distribution, and accumulation of metabolites. KEGG analysis further clarified that primary metabolic systems (sugars, amino acids, and lipids) were the locations of the differential pathways, particularly pyruvate metabolism, which serves as a precursor source for fatty acid synthesis, and protein processing, which affects the assembly of glycerolipids in the endoplasmic reticulum.

### 3.4. Expression Profiling of Genes Involved in the TG-Centered Lipid Metabolic Pathway

**Figure 3:**
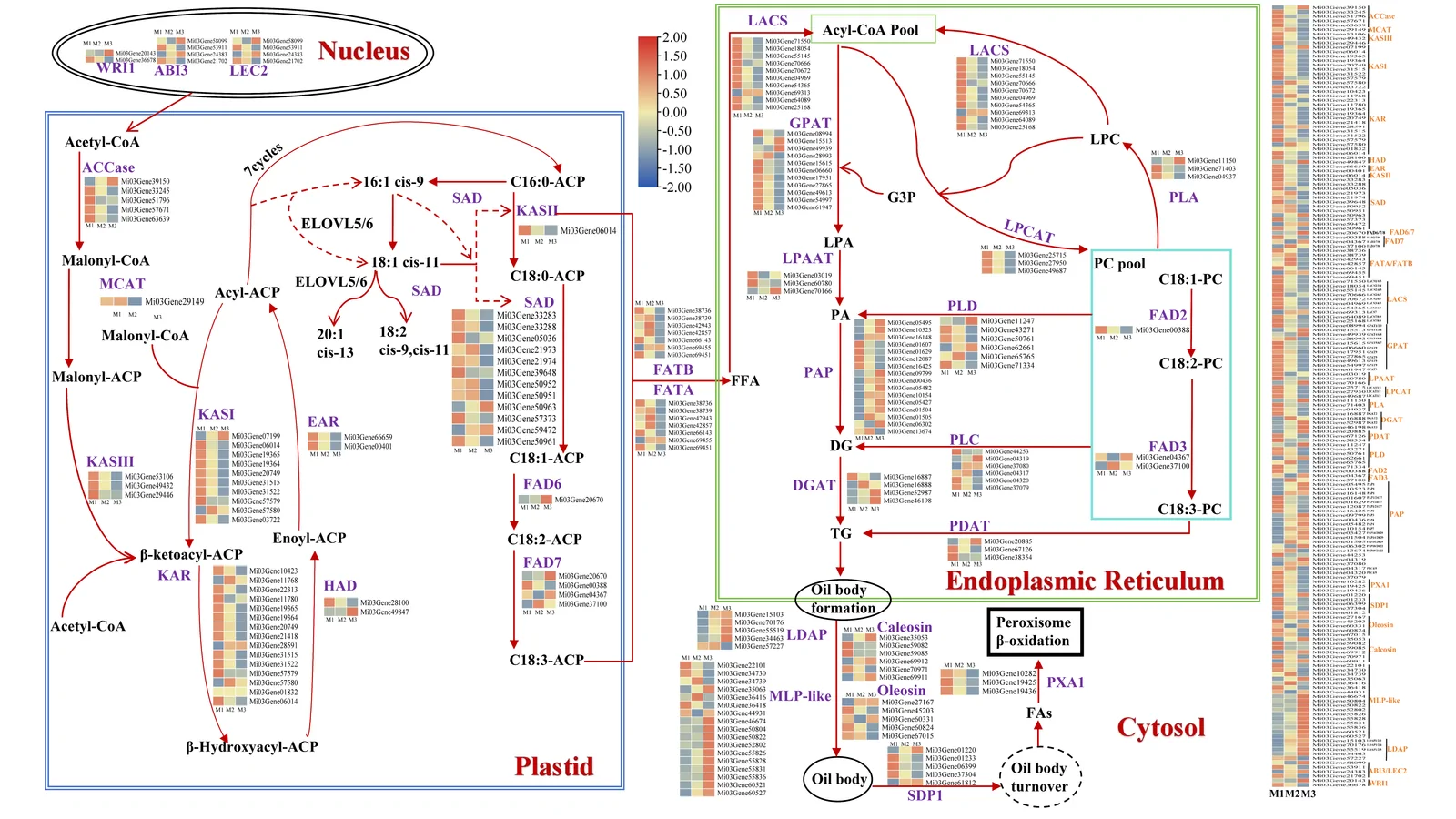
Hypothesized metabolic pathway differences for the core lipid TG in kernels of the three macadamia cultivars. Note: M1 denotes the comparison group DS1_S2 vs. GR_S2, M2 denotes the comparison group DS1_S2 vs. OC_S2, and M3 denotes the comparison group GR_S2 vs. OC_S2. The expression level of each gene shown in the figure was derived from FPKM values after log2 normalization and is presented as a heatmap to reflect upregulated (>1, in warm colors) and downregulated (<1, in cold colors) expression. Abbreviations: WRI1, WRINKLED1. ABI3, ABSCISIC ACID INSENSITIVE 3. LEC2, LEAFY COTYLEDON 2. Acetyl-CoA, Acetyl-Coenzyme A. ACCase, Acetyl-CoA Carboxylase. Malonyl-CoA, Malonyl-Coenzyme A. MCAT, Malonyl-CoA, ACP Transacylase. ACP, Acyl Carrier Protein. KASIII, 3-Ketoacyl-ACP Synthase III. KAR, Ketoacyl-ACP Reductase. HAD, Hydroxyacyl-ACP Dehydratase. EAR, Enoyl-ACP Reductase. KASI, 3-Ketoacyl-ACP Synthase I. KASII, 3-Ketoacyl-ACP Synthase II. SAD, Stearoyl-ACP Desaturase. FATA, Fatty Acyl-ACP Thioesterase A. FATB, Fatty Acyl-ACP Thioesterase B. FFA, Free Fatty Acid. LACS, Long-Chain Acyl-CoA Synthetase. Acyl-CoA Pool, Acyl-Coenzyme A Pool. FAD2, Delta-12 Fatty Acid Desaturase. FAD3, Delta-15 Fatty Acid Desaturase. FAD6, Omega-6 Fatty Acid Desaturase. FAD7, Omega-3 Fatty Acid Desaturase. ELOVL5, Elongation of Very Long Chain Fatty Acids Protein 5. ELOVL6, Elongation of Very Long Chain Fatty Acids Protein 6. GPAT, Glycerol-3-phosphate Acyltransferase. LPA, Lysophosphatidic Acid. LPAAT, Lysophosphatidic Acid Acyltransferase. PA, Phosphatidic Acid. PAP, Phosphatidic Acid Phosphatase. DGAT, Diacylglycerol Acyltransferase. PDAT, Phospholipid Diacylglycerol Acyltransferase. MLP-like protein, Major latex-like protein. LDAP, Lipid Droplet Associated Protein. PXA1, Peroxisomal ABC Transporter 1. LPCAT, Lysophosphatidylcholine Acyltransferase. PLD, Phospholipase D. PLC, Phospholipase C. SDP1, SUGAR-DEPENDENT 1. 16:1 cis-9, Palmitoleic Acid. 18:1 cis-11, cis-Oleic Acid. 18:2 cis-9, cis-11, Conjugated Linoleic Acid. 20:1 cis-11, 11-Eicosenoic Acid.

Based on the results of comparative transcriptomic analysis, combined with existing theoretical understanding of the regulatory mechanisms of plant lipid metabolism, the gene expression patterns and differences in the TG-centered lipid metabolic pathway of the S2 stage kernels of three macadamia cultivars were inferred. Figure 3 mainly illustrated the three fundamental stages of TG metabolism: de novo fatty acid biosynthesis in the plastid, storage lipid molecule assembly and oil body formation in the endoplasmic reticulum, and oil body degradation and β-oxidation in the cytoplasm. The structural genes associated with each stage have multiple single-gene transcripts, and their expression levels differed significantly among cultivars (Supplementary Table S4). At the initiation stage of fatty acid biosynthesis, significant interspecific expression differences were observed among the three key upstream transcription factors WRI1, ABI3, and LEC2, with multiple single-gene transcripts exhibiting differentiated expression patterns across the three comparison groups; some transcripts were significantly upregulated in DS1_S2 vs. GR_S2 but downregulated in GR_S2 vs. OC_S2, while others showed the opposite trend, suggesting that these transcription factors may contribute to different degrees in promoting fatty acid biosynthesis in macadamia kernels across different cultivars. In the subsequent acyl flux sequential catalysis process, important regulatory genes such as ACCase, KAS, KAR, SAD, and FAD also exhibited similar expression patterns; for example, among the five single-gene transcripts of ACCase, four were significantly upregulated in DS1 vs. GR but downregulated in GR_S2 vs. OC_S2.

In TG molecular assembly, DG normally serves as the direct substrate for TG synthesis, and among the 16 transcripts of the PAP gene that regulates its synthesis, most were downregulated in the DS1_S2 vs. GR_S2 comparison group but upregulated in GR_S2 vs. OC_S2. Similarly, DGAT and PDAT, the two core rate-limiting enzymes that directly catalyze TG production via the acyl-dependent and independent pathways, respectively, also showed similar differential expression patterns in their single-gene transcripts across the three comparison groups, acting in coordination with the PLD and FAD2 genes that regulate PC and the specific accumulation of oleic acid therein. During the subsequent oil body synthesis and degradation stages, MLP-like and LDAP were mainly upregulated in the GR_S2 vs. OC_S2 comparison group, whereas Oleosin and Caleosin were upregulated in the DS1 vs. GR comparison group, and the two lipases SDP1 and PXA1 were mainly upregulated in the DS1_S2 vs. GR_S2 comparison group. Apparently, TG metabolism in the three cultivars exhibited pronounced gene expression differences at the levels of synthesis, accumulation, and degradation, and the differential up- and downregulated expression of distinct single-gene transcripts of the same gene across cultivars reflects a gene expression preference that may be attributable to cultivar-associated differences.

### 3.5. qRT-PCR Validation of Key Lipid Metabolism Related Genes

**Figure 4:**
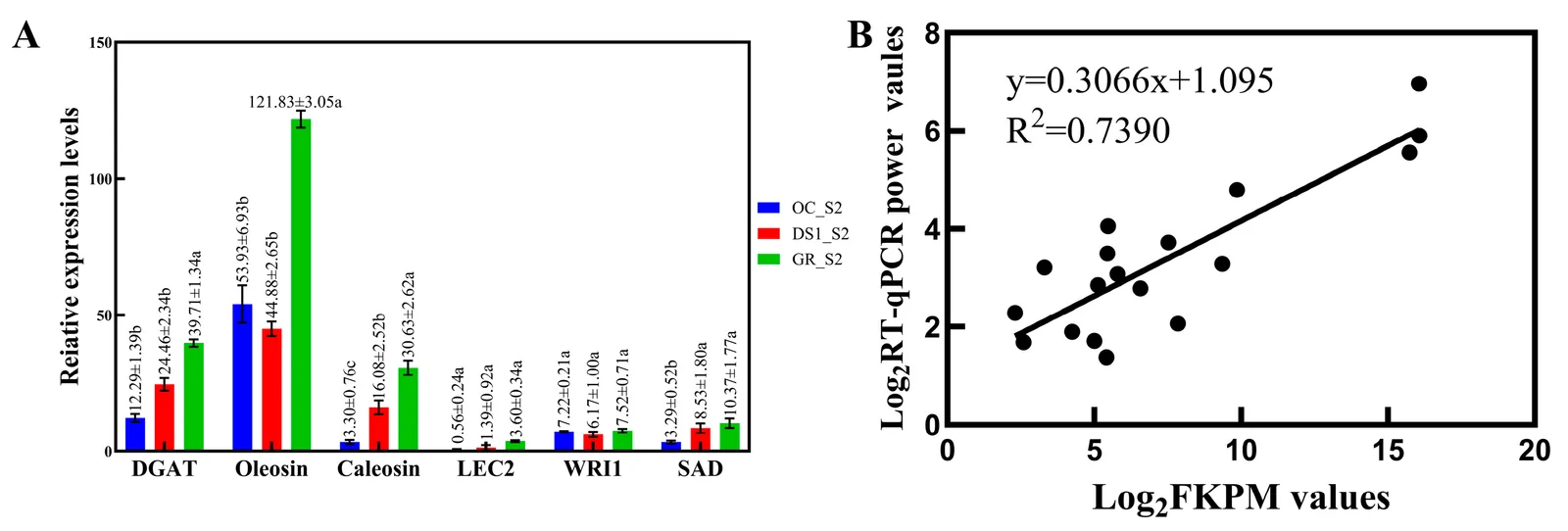
qRT-PCR validation of the expression levels of six key genes regulating lipid metabolism at the S2 stage. (**A**) Relative expression levels of six key lipid metabolism genes validated by qRT-PCR at the S2 stage. Data are presented as mean ± standard deviation (SD) with three biological replicates (*n* = 3). Different lowercase letters above the bars indicate significant differences among cultivars (*p* < 0.05, one-way ANOVA followed by LSD post-hoc test). (**B**) Correlation analysis between relative gene expression levels and their FPKM values in RNA-seq. Moderate positive linear correlation, R^2^ = 0.7390, *n* = 18 independent data points. Note: The single-gene transcript validated was the transcript with the highest FPKM value among all transcripts annotated for that gene in the transcriptomic analysis. Different letters above the bars indicate significant differences (*p* < 0.05) in the relative expression of each gene among different cultivars under the LSD test.

To validate the reliability of the transcriptomic data, we selected the single-gene transcripts of six key lipid metabolism genes with the highest FPKM values, including DGAT, Oleosin, Caleosin, LEC2, WRI1, and SAD, for qRT-PCR expression detection (Figure 4). The results showed that the relative expression levels of the transcripts of the six genes differed significantly overall among the three cultivars, with Oleosin showing the highest expression, followed by Caleosin and LEC2, while DGAT, WRI1, and SAD were relatively lower. Correlation analysis between the log_2_-normalized qRT-PCR expression levels and the FPKM values revealed a moderate positive linear correlation between the two (R^2^ = 0.7390, *n* = 18 data points), confirming the reliability of the RNA−seq results.

### 3.6. Comparative Metabolomic and Lipidomic Analysis Among Three Macadamia Cultivars at the S2 Stage

**Figure 5:**
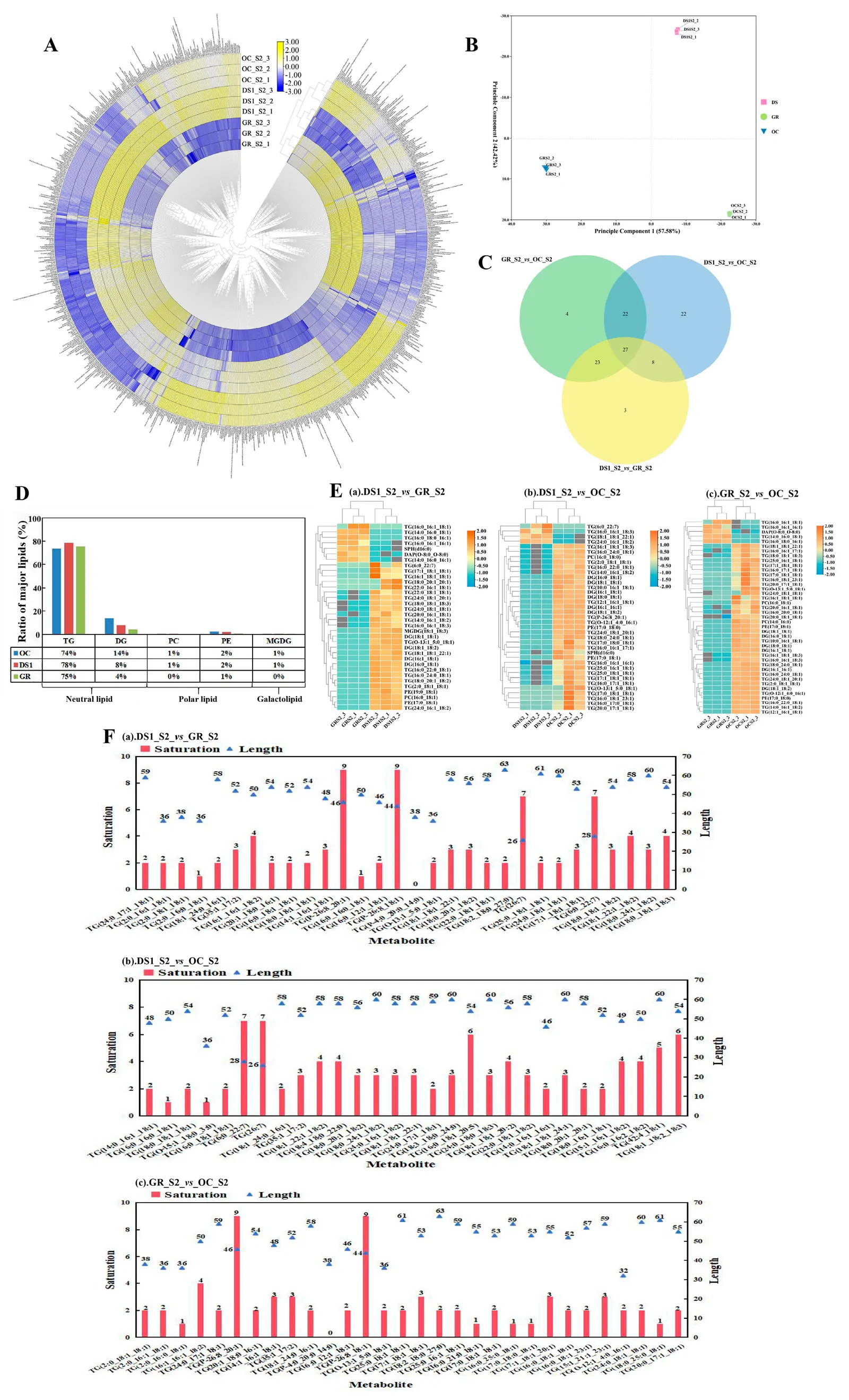
Comparative metabolomic and lipidomic analysis of metabolic profile differences in kernels at the S2 stage among three macadamia cultivars: (**A**) heatmap of total metabolite abundances; (**B**) PCA analysis; (**C**) Venn diagram analysis of DAMs; (**D**) comparison of lipid subclasses; (**E**) heatmap visualization of DAL abundance; (**F**) comparison of chain-length and saturation differences among the top 30 TG molecules with the highest relative contents. Note: In DAMs and DALs, warm colors (yellow, orange) indicate up-regulated accumulation, and cool colors (blue, light green) indicate down-regulated accumulation. Comparison of fatty acid chain length and saturation degree of the top 30 most abundant TG molecules. The *x*-axis indicates the total number of carbon atoms in the three fatty acyl chains of each TG molecule (chain length, a dimensionless count index). The *y*-axis indicates the total number of double bonds in the three fatty acyl chains of each TG molecule (saturation degree, a dimensionless count index).

As shown in Figure 5, metabolomic and lipidomic analysis were further employed to compare the metabolic profile differences among the kernels of the three macadamia cultivars at the S2 stage. Metabolomic analysis detected a total of 824 metabolites, which were classified into 15 categories, with relative abundances in descending order as organooxygen compounds (19.27%), carboxylic acids and derivatives (15.78%), prenol lipids (13.97%), fatty acyls (7.26%), cinnamic acids and derivatives (3.35%), flavonoids (3.21%), and the others in lower proportions (Supplementary Table S2). The accumulation abundances of these metabolites differed significantly among the three cultivars (Figure 5A). PCA analysis showed that the three cultivars formed independent clusters along the first principal component (PC1, 45.06%), with small intragroup variation and clear intergroup separation, indicating pronounced cultivar specificity in metabolite synthesis and accumulation (Figure 5B). A total of 216 DAMs were identified, comprising 61 (30 upregulated and 31 downregulated) in the DS1_S2 vs. GR_S2 comparison, 79 (39 upregulated and 40 downregulated) in the DS1_S2 vs. OC_S2 comparison, and 76 (37 upregulated and 39 downregulated) in the GR_S2 vs. OC_S2 comparison, with 27 DAMs shared among all three comparison groups (Figure 5C).

Likewise, lipidomic analysis annotated a total of 304 lipid molecular species belonging to 24 lipid classes. When calculated based on the count of annotated molecular species, TG represented the largest class and accounted for 53.69% of all annotated lipid molecular species, followed by DG (11.07%), while the remaining classes, including PC, DGDG (digalactosyldiacylglycerol), and PE, each accounted for less than 5% (Supplementary Table S5). In general, the lipid subclass differences among the three cultivars were concentrated in neutral storage lipids, with TG and DG being the major contributing variables (Figure 5D), a finding consistent with the quality analysis results (Figure 1C). A total of 112 DALs were identified, distributed across the three comparison groups as follows: 34 (DS1_S2 vs. GR_S2), 37 (DS1_S2 vs. OC_S2), and 41 (GR_S2 vs. OC_S2), with most concentrated in TG. The heatmap revealed a pronounced cultivar-specific pattern in DAL accumulation (Figure 5E), in particular, OC_S2 tended to accumulate more high carbon chain TG molecules containing MUFAs and LCFAs, such as TG (24:0_18:1_20:1) (putatively annotated) and TG (25:0_16:1_18:1) (putatively annotated), whereas DS1_S2 exhibited significantly greater accumulation of TG molecules with relatively higher fatty acid saturation, such as TG (18:0_18:0_18:1) (putatively annotated) and TG (16:0_18:0_18:1) (putatively annotated). Analysis of chain length and saturation revealed that the total fatty acid chain lengths of the top 30 most abundant TG molecules ranged from 50 to 60 carbon atoms, with saturation levels fluctuating between 0 and 9. This fluctuation was particularly pronounced in the DS1_S2 vs. GR_S2 comparison, while the other two comparison groups remained relatively stable (Figure 5F). Together, these findings directly reflected the complex influence of differential gene expression on kernel quality formation in the three examined macadamia cultivars at the S2 stage.

### 3.7. Variation in DAMs Among the Three Macadamic Cultivars at the S2 Stage

Supplementary Table S6 further analyzed the specific variations of the 216 DAMs across the three comparison groups. It is obvious that the GR_S2 vs. OC_S2 comparison group exhibited the most pronounced fluctuations, in which Icariside D2 (putatively annotated) and Melilotoside (putatively annotated) (FC: 201.29 and 120.08) in the Organooxygen compounds category, Ajugol (putatively annotated) (FC: 126.46) in the Prenol lipids category, and Tenuifoliside B (putatively annotated) (FC: 711.71) in the Cinnamic acids and derivatives category showed FC values that were dozens to hundreds of times higher than those of other metabolites of the same category and those in the other two comparison groups. Differences in the DS1_S2 vs. OC_S2 comparison group were concentrated in Organooxygen compounds and Purine nucleotides, with FC values also showing significant variation, whereas in the DS1_S2 vs. GR_S2 comparison group, apart from the extremely high FC value of 6-Hydroxy-4-methylcoumarin (putatively annotated) (1024), the remaining changes were relatively balanced, with only a small number of metabolites such as Rubinaphthin A (putatively annotated), UDP-D-Xylose (putatively annotated), and N-Acetyl-L-glutamic acid (putatively annotated) showing fluctuations of around dozens of times. This further demonstrated the relatively pronounced differences in primary and secondary metabolic differentiation characteristics of kernels among the three macadamia cultivars DS1, GR, and OC at the S2 stage.

### 3.8. Integrated Omics Analysis on Carbon Metabolism and Gene-Lipid Interactions

**Figure 6:**
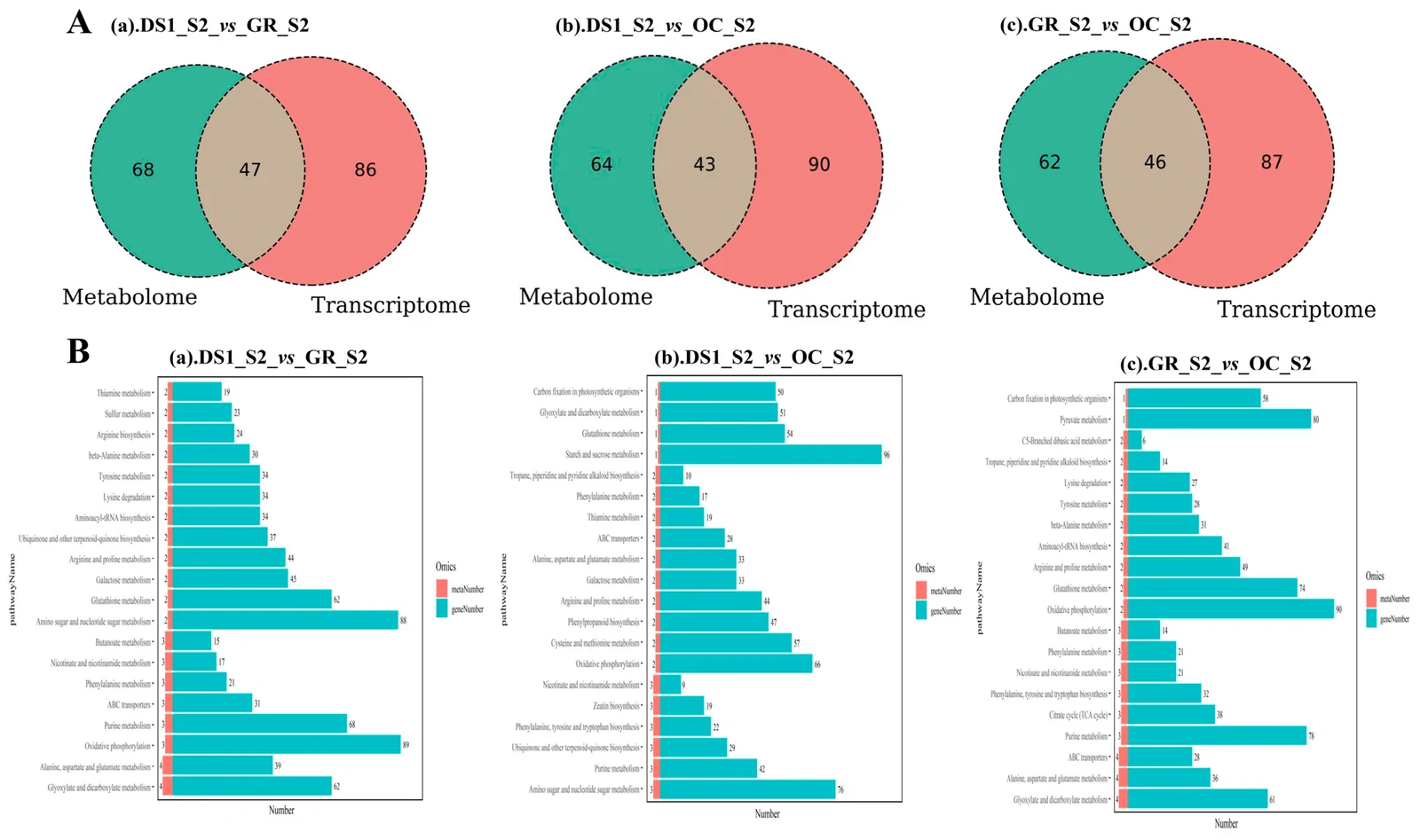
Multi-omics joint analysis. (**A**) Venn diagram (DEGs-DAMs). (**B**) Top 10 KEGG pathways (DEGs-DAMs). Note: (**A**) Venn diagrams showing the overlap between DEGs and DAMs for each pairwise cultivar comparison. (**B**) Top-10 enriched KEGG pathways based on combined DEG-DAM datasets.

Finally, a joint analysis was performed on the metabolic pathways involved in the DEGs and DAMs/DALs identified in the three macadamia cultivars. Figure 6A showed that the metabolic pathways involved in DEGs and DAMs across the three comparison groups were generally similar in distribution and shared numbers, suggesting that although differences in gene expression and metabolite accumulation at the S2 stage were significant among cultivars, the range of pathways involved was relatively concentrated. KEGG enrichment analysis revealed hierarchical differential regulation of core carbon metabolism pathways among the three comparison groups (Figure 6B), with glycolysis/gluconeogenesis and starch and sucrose metabolism being the mutually core enriched pathways. The pathway response intensity ranked as GR_S2 vs. OC_S2 > DS1_S2 vs. GR_S2 > DS1_S2 vs. OC_S2. Among these, GR_S2 vs. OC_S2 exhibited the highest number of differentially expressed molecules, with glycolysis, TCA cycle, and glyoxylate metabolism responding synchronously, indicating that the differences in carbon skeleton conversion and organic acid distribution between GR and OC were the greatest. In DS1_S2 vs. GR_S2, starch and sucrose metabolism and thiamine metabolism were significantly enriched, indicating pronounced transcriptional differentiation in carbohydrate degradation and cofactor metabolism between them. In DS1_S2 vs. OC_S2, carbon fixation, pyruvate metabolism, and phenylpropanoid biosynthesis pathways were more active, reflecting the relatively distinctive characteristics of OC in photosynthetic product allocation and secondary metabolic flux. In summary, the intensity of carbon metabolic regulation among the three macadamia cultivars showed a stepwise differentiation, with the greatest metabolic divergence between GR and OC, and DS1 representing an intermediate transitional type.

Integrated multi-omics analysis suggested that phenotypic differences in kernel quality among the three macadamia genotypes in the S2 stage were jointly driven at both transcriptional and metabolic levels, with pronounced differentiation in carbon-source utilization strategies associated with differences in gene expression. Integrated multi-omics analysis suggested that phenotypic differences in kernel quality among the three macadamia genotypes in the S2 stage were jointly driven at both transcriptional and metabolic levels, with pronounced differentiation in carbon-source utilization strategies associated with differences in gene expression.

## 4. Discussion

This study focused on three representative macadamia cultivars, DS1, GR, and OC, grown in the hot and dry valley region of Dehong, Yunnan, and comprehensively analyzed the changes in key quality components of the kernel from the young fruit stage to the mature stage. Targeting the S2 stage, a critical metabolic transition node, a multi-omics joint analysis strategy was applied to dissect the lipid metabolism regulatory network centered on TG, thereby identifying molecular patterns and candidate genes associated with differential lipid accumulation among cultivars.

Spatiotemporal dynamic analysis of macadamia kernel quality components showed that although the three tested cultivars exhibited significant differences in quality characteristics, they shared the same developmental trends. The total lipid content continued to increase with developmental progression in all cultivars, reaching a peak at the S3 stage, whereas the total protein and fiber contents decreased continuously in parallel, and the total soluble sugar content exhibited a trend of initially increasing and then decreasing. Though the overall starch content was relatively low, its concentration fluctuations were observed to parallel the changes in total soluble sugars across developmental stages. It is worth noting that only phenotypic concentration data for starch are available in this work; formal statistical comparisons of starch dynamics, activity assays of key starch metabolic enzymes such as ADP-glucose pyrophosphorylase (AGPase), starch synthase (SS), starch branching enzyme (SBE), and targeted gene expression analysis were not conducted. From this phenotypic covariation alone, we raise the plausible observational hypothesis that starch may serve as a transient carbon reservoir for temporary carbon−skeleton storage and mobilization before rapid lipid accumulation in macadamia kernels, but this theory awaits further mechanistic experimental validation [41]. This sequential conversion pattern among carbon assimilation products is similar to the metabolic redistribution patterns observed during seed development in woody oil crops such as *Camellia oleifera* Abel [42] and oil palm (*Elaeis guineensis* Jacq) [43]. Fatty acid analysis simultaneously revealed the transformation pattern of major acyl fluxes, with the continuous decline in palmitoleic acid proportion accompanied by a continuous increase in oleic acid proportion representing the most notable feature. This pattern suggests that the biosynthesis of these two MUFAs may involve substrate competition or a potential shift in desaturase activity as fruit development proceeds, a phenomenon similarly reported in avocado (*Persea americana* Mill.) [44]. Among the three developmental stages, S2 was identified as a critical inflection point for quality differentiation, with total lipid content exhibiting its largest increase during this stage; for instance, the total lipid content of GR surged from 27.94% at S1 to 50.16% at S2, an increase of nearly two-fold. Concurrently, protein and fiber contents declined sharply by more than two-fold, while total soluble sugar content also peaked during this period. These observations show that the S2 stage is a period of substantial carbon-metabolism rearrangement in macadamia kernels. It may also represent a key developmental window when primary metabolism shifts toward lipid biosynthesis. These cultivar- and stage-specific differences provide clear phenotypic guidance for subsequent elucidation of the underlying molecular basis.

Comparative transcriptomic analysis was first performed to identify transcriptional regulatory differences among the three cultivars during the S2 stage. PCA confirmed that gene expression profiles were highly cultivar-specific. GO enrichment analysis revealed that DEGs were mainly concentrated in cellular component categories related to metabolic sites, such as intracellular anatomical structure and intracellular/membrane-bounded organelles, which was highly consistent with the subcellular structural remodeling occurring during S2, a stage characterized by extensive oil body formation and rapid lipid accumulation in kernel cells [7]. KEGG pathway analysis further pointed to core steps of primary carbon assimilation metabolism, including glycolysis/gluconeogenesis, pyruvate metabolism, and protein processing in the endoplasmic reticulum. These pathways represent key upstream steps that supply carbon skeleton precursors, such as acetyl-CoA, for de novo fatty acid synthesis and ensure the proper folding and processing of storage proteins and lipid synthesis enzymes [45,46]. Based on the transcriptomic data, we integrated the fundamental and classical metabolic pathways of TG in S2-stage kernel cells, spanning plastidial fatty acid synthesis, endoplasmic reticulum assembly, and oil body formation and degradation. Pathway inference results indicated that multiple single-gene transcripts of upstream transcription factors such as WRI1, ABI3, and LEC2 exhibited significant expression differences among varieties, and this variation was propagated downstream throughout the entire fatty acid synthesis system, with acyl-flux control genes including ACCase, KAS, KAR, and HAD displaying largely consistent differential expression patterns across the three comparison groups. In the TG assembly stage, the PAP gene, which influences DG synthesis, along with DGAT and PDAT, the two key rate-limiting enzymes of the Kennedy pathway and the Lands cycle, similarly showed comparable patterns of variation, which is consistent with the proposed contribution of these transcription factors to lipid metabolism [47,48]. Notably, the key regulatory metabolic node governing oleic acid turnover exhibited clear transcriptional differences among cultivars. The FAD2 gene, which encodes oleate desaturase, showed higher transcriptional abundance in DS1, the cultivar with the lowest oleic acid content, a pattern consistent with its function catalyzing the conversion of C18:1 to C18:2, which could partially account for DS1’s phenotypic characteristic of relatively low oleic acid accumulation coupled with a higher linoleic acid proportion. In contrast, comparatively lower FAD2 transcript levels in OC and GR may limit further desaturation of oleic acid and favor its net accumulation, which aligns with their higher oleic acid content phenotypes (from S2 to S3 stages: OC 60.08–61.77%, GR 55.13–64.96%, respectively). Nevertheless, this interpretation remains correlative and tentative. Quantitative transcript values for FAD2 are not explicitly presented herein, and direct FAD2 enzymatic-activity measurements were not performed in this study. Other fatty acid desaturase coding genes (e.g., FAD3, FAD6, and FAD7) also contribute to fatty acid profile remodeling, yet their individual functional contributions were not assessed in this transcript-level comparison. Moreover, oleic acid net accumulation may also be shaped by upstream carbon anabolic pathway flux, the availability of the C18:1-ACP substrate pool, or the activity of acyl-ACP thioesterases (FatA/FatB). Thus, it cannot be ruled out that factors independent of FAD2 transcriptional variation constitute major drivers of cultivar-specific oleic acid phenotypic divergence. Furthermore, the PLD gene, which is involved in membrane lipid remodeling, was upregulated in the DS1 vs. GR comparison group and may indirectly regulate the composition of the acyl pool by affecting the turnover of membrane lipids such as PC, although it does not directly catalyze oleic acid production. The differential expression of these key node genes, which collectively represent molecular correlates associated with fatty acid profile divergence among cultivars, centers on the ratio of oleic acid to linoleic acid. The oleosin gene family influences the homeostasis of TG storage; among these genes, Oleosin and Caleosin were upregulated in the DS1 vs. GR comparison group, whereas MLP-like and LDAP were upregulated in the GR vs. OC comparison group, suggesting that different cultivars may favor distinct strategies for oil body assembly and stabilization. This difference in oleosin composition may affect the stability and size of lipid droplets [49,50], which in turn may be associated with differences in oil accumulation rate and final oil content among cultivars. With respect to lipid degradation, the lipolysis genes SDP1 and PXA1 were significantly upregulated in the DS1_S2 vs. GR_S2 comparison group, suggesting that the vigorous lipid accumulation observed in DS1_S2 at the S2 stage, relative to GR, may be accompanied by a more active lipid turnover process. This transcriptional signature was consistent with the phenotypic trend observed in DS1, wherein although the total lipid content at S1 was relatively high (39.51%), the oil accumulation rate from S2 to S3 and the final oil content were both lower than those of GR, suggesting that the synthesis-degradation dynamic balance of lipids in DS1 may favor a faster turnover rate, thereby limiting the efficiency of net oil accumulation.

Furthermore, plant hormone-mediated regulatory networks exert profound upstream control over seed storage lipid biosynthesis, acting upstream of master transcription factors including WRI1, ABI3 and LEC2. Key hormone metabolism related genes, such as 9-cis-epoxycarotenoid dioxygenase (NCED) and (+)-abscisic acid 8′-hydroxylase (CYP707A) for abscisic acid (ABA) biosynthesis and catabolism, gibberellin 20-oxidase (GA20ox) and gibberellin 3-oxidase (GA3ox) for gibberellin (GA) biosynthesis, 1-aminocyclopropane-1-carboxylate oxidase (ACO) for ethylene production, and auxin response factor (ARF) for auxin signal transduction, coordinate kernel development and carbon flux allocation toward TG accumulation in oil rich seeds [51,52]. ABA promotes seed maturation and oil deposition, while bioactive GAs modulate cell expansion and can antagonize ABA-driven maturation programs; auxin and ethylene further fine-tune these developmental outputs via transcriptional crosstalk with LEC type and WRI1 family regulators [53,54]. Although our transcriptomic dataset captured variation in these hormone-related gene transcripts across cultivars at S2, the present multi-omics experimental design prioritized the downstream lipid metabolic cascade. Thus, it may not be able to draw definitive causal conclusions linking cultivar-associated kernel quality divergence to hormone level shifts. Future targeted transcript, hormone, and metabolite combined profiling will be required to untangle how ABA, GA, auxin and ethylene signaling modulate lipid metabolism transcription factor activity in developing macadamia kernels [55]. It should be explicitly noted that phytohormone contents were not directly quantified in the present study. Although our transcriptome dataset contains sequence information for hormone-related genes, the targeted differential expression analysis or functional interpretation for this set of hormone-related genes was not performed. Any links between hormone signaling and cultivar-associated kernel quality patterns remain speculative. Hence, direct hormone profiling together with dedicated gene set enrichment shall be needed in future work to verify hormone-dependent regulatory effects in macadamia kernel quality development.

Differences in starch and sugar-related carbohydrate pathways (Supplementary Figure S1 and Table S7) offered important clues about upstream carbon supply linked to variable lipid accumulation. For example, hexokinase (HXK) catalyzes the irreversible rate-limiting step that produces glucose-6-phosphate in sugar metabolism [56]. Seven single-gene transcripts of HXK were significantly differentially expressed in the DS1_S2 vs. GR_S2 and GR_S2 vs. OC_S2 comparison groups, indicating that the initial carbon source activation rate diverged among varieties. Ten transcripts of PFK (phosphofructokinase), the core rate-limiting enzyme of glycolysis downstream, exhibited completely opposite expression patterns in these two comparison groups, and some transcripts were not significantly differentially expressed in DS1_S2 vs. OC_S2. This fine-scale transcriptional regulatory difference is evidently associated with differences in carbon-related metabolic patterns toward pyruvate and acetyl-CoA, thereby affecting the precursor supply for de novo fatty acid synthesis [57]. Furthermore, key genes catalyzing anaplerotic reactions and transmembrane shuttling of metabolites, such as PEPC (phosphoenolpyruvate carboxylase), PPDK (pyruvate phosphate dikinase), and TPI (triose phosphate isomerase), also exhibited consistent differential expression patterns across the comparison groups, suggesting that carbon flux partitioning from carbohydrate metabolism to lipid synthesis was subject to global transcriptional regulation. Subsequent qRT-PCR validation of six key genes, namely DGAT, Oleosin, Caleosin, LEC2, WRI1, and SAD, confirmed the reliability of the transcriptomic data and substantiated the active involvement of these genes in the lipid metabolism system of macadamia kernels. Notably, during the S3 stage, Oleosin and DGAT maintained relatively high transcript levels while the expression of the other genes declined overall (Supplementary Figure S2), indicating that the terminal catalytic step of TG synthesis and the oil body assembly process remain the core steps sustaining lipid accumulation in kernels when entering the maturation stage. This finding is consistent with the physiological pattern of oilseed development transitioning from active synthesis to mature storage [58,59,60].

Comparative metabolomic and lipidomic analysis elucidated, at the level of metabolite accumulation, the actual effects arising from differences in transcriptional regulation. A total of 824 metabolites were identified in the metabolome, and the PCA results were highly consistent with those of the transcriptome, confirming the metabolic specificity of the tested cultivars from a phenotypic perspective. Regarding the FC distribution of the 216 DAMs identified through screening, the GR_S2 vs. OC_S2 comparison group exhibited the most pronounced metabolic differences, with several metabolites belonging to different chemical classes, such as Icariside D2 (putatively annotated) and Melilotoside (putatively annotated), showing levels several orders of magnitude higher than their counterparts in other comparison groups. This suggests that the two cultivars may exhibit nodal, extreme metabolic divergence at certain stages of primary and secondary metabolism. In contrast, the metabolic differences in the DS1_S2 vs. GR_S2 comparison group were relatively balanced, with only a small number of metabolites, such as 6-hydroxy-4-methylcoumarin (putatively annotated), showing extreme values. This distribution pattern indicates that although all three cultivars displayed evident metabolic specificity at the S2 stage, the degree of divergence was not uniform. The metabolic differences between OC and GR were far greater than those between OC and DS1, which may be related to the unique genetic background of OC and its ecological adaptability. Lipidomic analysis identified TG as the absolute core component of the S2 stage kernel lipidome, accounting for as much as 53.69%, followed by DG at 11.07%. This compositional feature was consistent with the functional characterization of the S2 stage as a period of rapid storage lipid accumulation in the kernel. Notably, DG, as the direct precursor of TG synthesis, exhibited significant differences in relative abundance among the three cultivars (OC 14% > DS1 8% > GR 4%). DG abundance is closely associated with the TG synthesis rate; greater DG accumulation generally suggests a potentially lower conversion efficiency at the terminal acylation step [61]. This inference was compared against total lipid phenotypic data obtained from Figure 1C; the GR cultivar had a high total lipid content (50.16%) coupled with the lowest DG residue (4%) at the S2 stage, reflecting its efficient conversion capacity from DG to TG. Although OC possessed the highest total lipid content (54.21%) at S2, it retained the highest DG proportion (14%), implying a potential bottleneck in terminal acylation that limits complete conversion of DG towards TG despite robust overall lipid biosynthesis. DS1 occupied an intermediate status, with a total lipid content of 43.06% and DG residue of 8%. Cluster analysis of DALs further revealed cultivar-specific preferences in the accumulation of particular lipid molecules. Specifically, OC tended to favor the enrichment of TG molecules containing MUFAs and LCFAs, such as TG (24:0_18:1_20:1) (putatively annotated), whereas DS1 and GR displayed more diversified TG accumulation profiles. This finding was consistent with the analysis of differences in total fatty acid chain length and saturation among the top 30 most abundant TG molecules, reflecting cultivar-specific differences in the expression activity of fatty acid elongases and desaturases, which may substantially influence oil oxidative stability, nutritional quality, and the precise industrial positioning of each cultivar [62,63].

At last, the multi-omics integrated analysis revealed the molecular associations underlying kernel quality differences among the macadamia varieties from the perspective of metabolic pathway coordination. Among the relatively stable differential or shared metabolic pathways, energy and carbon skeleton supplying pathways, including oxidative phosphorylation, glyoxylate and dicarboxylate metabolism, and the TCA cycle, were significantly enriched across each cultivar, indicating that the core differences in kernel quality involve not only the lipid metabolism system but also differences in upstream carbon flux allocation and energy supply efficiency. Specifically, oxidative phosphorylation represents mitochondrial ATP supply capacity [64], glyoxylate metabolism is linked to the TCA cycle through the supply of acetyl-CoA precursors [65], and glutathione metabolism helps maintain cellular redox balance [66]. The coordinated changes in these pathways collectively shaped the differential phenotypes of carbon assimilation and accumulation in the kernels of the three tested cultivars during the S2 stage. It is worth noting that the pathways discussed above corroborate the hierarchical carbon metabolism regulatory features revealed by the joint KEGG enrichment analysis (Figure 6B). Glycolysis/gluconeogenesis and starch and sucrose metabolism, serving as hub pathways for carbon flux allocation, exhibited peak response intensity in the GR_S2 vs. OC_S2 comparison group, accompanied by the concurrent activation of the TCA cycle and glyoxylate metabolism, which directly reflects the extent of metabolic rearrangement potentially linked to precursor supply for lipid biosynthesis. This gradient distribution indicated that the carbon source utilization strategies of the different cultivars had diverged markedly by the S2 stage. GR and OC represent two typical modes, namely, carbon flux efficiently directed toward lipid synthesis and carbon flux maintained in relative balance between primary and secondary metabolism, respectively, whereas DS1 occupies an intermediate transitional state between the two. Therefore, the differences in lipid accumulation efficiency among varieties could be partially attributed, at the level of coordinated metabolic pathway responses, to fundamental differences in their carbon allocation logic.

Last but not least, it would be important to state a key limitation of the present study. Although physiological and biochemical phenotypic data were collected from three developmental stages, the multi-omics profiling was exclusively performed at the S2 stage. We selected S2 because phenotypic evidence demonstrated that S2 represents the critical metabolic turning point, accompanied by massive carbon re-allocation, and genotypic differences among DS1, GR and OC become most pronounced at this developmental phase. This experimental design mainly focuses on genotypic molecular divergence at such a switch-like stage rather than tracing intra-genotype developmental dynamics. Therefore, our multi-omics data represent a molecular snapshot at the S2 stage. Future research shall be carried out to generate a more detailed temporal and spatial blueprint including multiple developmental stages, to systematically resolve the dynamic shifts of gene expression and metabolite accumulation throughout macadamia kernel development.

Furthermore, FDR multiple-testing correction was only applied for transcriptomic DEG identification in this work. DAMs and DALs from untargeted metabolomics and lipidomics were filtered by raw *p*-values combined with VIP and fold-change thresholds. Although multi-dimensional filtering can partially reduce false-positive signals, future studies should adopt FDR-adjusted *p*-values for metabolomic and lipidomic datasets for more stringent differential screening. Plus, it should also be mentioned that the current study mainly relies on multi-omics comparative analysis and qRT-PCR validation at the transcript level. Given post-transcriptional and post-translational regulatory effects, transcript abundance cannot fully reflect the actual catalytic activity of key lipid metabolic enzymes such as DGAT, PDAT, FAD2 and SAD. Direct enzyme activity measurements and ^13^C/^14^C isotope-labeling experiments should be taken into account afterward. These biochemical assays will be more convincing to further unravel the underlying biochemical mechanisms of fatty acid and TG metabolism in macadamia kernels. Additionally, qRT-PCR in this work only validated transcriptional expression patterns of the identified candidate genes; functional characterization, including in vivo and in vitro microorganism and plant assays, can provide more solid evidence. In the meantime, promoter sequences of pivotal lipid metabolism regulators such as WRI1, ABI3, and LEC2 transcriptional factors must be investigated as well [67], which were inferred from multi-omics correlation and expression profiling, rather than confirmed by biochemical or genetic evidence. Overall, direct functional validation of these candidate genes, promoter dissection and upstream regulatory transcription factors warrant important directions for follow-up investigations.

Another important experimental limitation of this study should be highlighted. All three biological replicates for each cultivar were collected from a single individual tree. As a result, inter-tree variation within each cultivar was not captured in our sampling scheme. The observed phenotypic and molecular differences among DS1, GR1 and O.C. are therefore cultivar-associated patterns rather than definitively proven genotypic effects. Variation may also originate from within-tree physiological micro-environmental effects. Consequently, the generalizability of our conclusions to broader populations of these cultivars requires further validation using multiple independent trees per cultivar in future investigations.

## 5. Conclusions

In summary, this study has conducted a multi-omics analysis of three distinct macadamia cultivars at the S2 stage, a critical physiological turning point in kernel quality formation. We identified candidate genes and metabolic patterns associated with genotypic differences in oil-related traits. Our results suggested that cultivar-associated differences in TG metabolism among the sampled trees are linked to differential expression of TG biosynthesis-related genes, as well as coordinated changes in primary and secondary carbon anabolic pathways. These observational associations require further functional validation and independent multi-tree sampling to establish robust genotypic regulatory relationships. Our findings may offer a certain reference value for deepening the understanding of the molecular regulatory mechanisms of lipid metabolism in macadamia, as well as for quality innovation and industrial application development. Future studies should further conduct detailed validation analysis of these identified key regulatory factors and elucidate their interaction effects from multiple dimensions, as well as their associations with the biosynthesis of important functionally active substances such as oleic acid and palmitoleic acid. For another, further exploration of the effects of gene-environment interactions on macadamia nut developmental quality under different ecological conditions may provide a more comprehensive scientific basis for precision cultivation, interspecific hybridization and complementation, and molecular breeding for quality improvement in macadamia-producing regions across Yunnan and the world.

## Figures and Tables

**Table 1:** Comparison of the fatty acid profile (%) in kernels of three macadamia cultivars.

Sample	<C16:0	C16:0	C16:1	C18:0	C18:1	C18:2	C18:3	≥C20
DS1_S1	1.03 ± 0.00 a	7.72 ± 0.01 a	38.34 ± 0.00 a	4.74 ± 0.01 a	37.27 ± 0.04 c	4.62 ± 0.02 a	0.53 ± 0.01 a	5.75 ± 0.02 a
DS1_S2	0.88 ± 0.00 b	5.30 ± 0.00 b	31.53 ± 0.03 b	4.18 ± 0.01 a	50.19 ± 0.08 b	2.40 ± 0.01 b	0.24 ± 0.00 b	5.28 ± 0.01 a
DS1_S3	0.67 ± 0.00 b	5.42 ± 0.01 b	21.61 ± 0.02 c	4.03 ± 0.01 a	60.77 ± 0.12 a	1.53 ± 0.03 c	0.16 ± 0.01 c	5.81 ± 0.02 a
GR_S1	2.16 ± 0.00 a	13.17 ± 0.00 a	22.39 ± 0.01 a	15.60 ± 0.00 a	35.72 ± 0.06 c	4.15 ± 0.01 a	0.95 ± 0.00 a	5.86 ± 0.02 b
GR_S2	0.96 ± 0.03 b	5.24 ± 0.02 b	20.45 ± 0.02 b	8.87 ± 0.02 b	55.13 ± 0.26 b	2.57 ± 0.11 b	0.11 ± 0.07 b	6.67 ± 0.01 a
GR_S3	0.33 ± 0.00 b	4.92 ± 0.02 c	19.21 ± 0.05 c	3.84 ± 0.01 c	64.96 ± 0.04 a	1.57 ± 0.01 c	0.15 ± 0.00 b	5.02 ± 0.01 b
OC_S1	1.26 ± 0.00 a	10.77 ± 0.00 a	34.39 ± 0.06 a	9.24 ± 0.01 a	35.86 ± 0.03 b	3.29 ± 0.00 a	0.83 ± 0.03 a	4.36 ± 0.03 b
OC_S2	0.87 ± 0.00 b	4.72 ± 0.01 c	23.89 ± 0.04 b	3.97 ± 0.01 c	60.08 ± 0.05 a	1.16 ± 0.02 b	0.15 ± 0.00 b	5.16 ± 0.01 a
OC_S3	0.69 ± 0.00 b	5.56 ± 0.01 b	21.68 ± 0.09 c	4.64 ± 0.01 b	61.77 ± 0.09 a	0.92 ± 0.00 c	0.18 ± 0.00 b	4.56 ± 0.01 b

Note: Different letters following the values indicate significant differences (*p* < 0.05) in the content of the same fatty acid across different growth stages within the same variety, as determined by the LSD test. All values are expressed as % of dry weight.

## Data Availability

The original contributions presented in the study are included in the article/Supplementary Materials; further inquiries can be directed to the corresponding author. The RNA-seq datasets generated and/or analyzed during the current study are available in the National Center for Biotechnology Information repository, [ACCESSION NUMBER: PRJNA1520119].

## Supplementary Materials

The following supporting information can be downloaded at: https://www.mdpi.com/article/10.3390/foods15213830/s1, Supplementary Figure S1: Hypothesized metabolic pathway differences in starch and sucrose metabolism in kernels of the three macadamia cultivars. Note: M1 denotes the comparison group DS1 vs. GR; M2 denotes the comparison group DS1 vs. OC; and M3 denotes the comparison group GR vs. OC. The expression level of each gene shown in the figure was derived from FPKM values after log2 normalization and is presented as a heatmap to reflect upregulated (>1, in warm color) and downregulated (<1, in cold color) expression. Abbreviations: UDP-Glu, uridine diphosphate glucose. Glu, glucose. Fru, fructose. G1P, glucose-1-phosphate. G6P, glucose-6-phosphate. F6P, fructose-6-phosphate. F1,6P, fructose-1,6-bisphosphate. GAP, glyceraldehyde-3-phosphate. 1,3-BPG, 1,3-bisphosphoglycerate. 3-PGA, 3-phosphoglycerate. 2-PGA, 2-phosphoglycerate. PEP, phosphoenolpyruvate. Pyr, pyruvate. Malate, malate. OAA, oxaloacetate. SUS, sucrose synthase. CWINV, cell wall invertase. CINV, cytosolic invertase. UGP, UDP-glucose pyrophosphorylase. PGM, phosphoglucomutase. GPI, glucose-6-phosphate isomerase. PFP, pyrophosphate-dependent phosphofructokinase. PFK, ATP-dependent phosphofructokinase. FBA, fructose-bisphosphate aldolase. GAPDH, glyceraldehyde-3-phosphate dehydrogenase. PGK, phosphoglycerate kinase. PGAM, phosphoglycerate mutase. ENO, enolase. PEPC, phosphoenolpyruvate carboxylase. PK, pyruvate kinase. ME, malic enzyme. MDH, malate dehydrogenase. PEPCK, phosphoenolpyruvate carboxykinase. TPI, triose phosphate isomerase. HXK, hexokinase. FK, fructokinase. GLT1, glucose transporter 1. GPT, glucose-6-phosphate/phosphate translocator. TPT, triose phosphate translocator. BASS, bile acid:sodium symporter family protein. DiT, dicarboxylate transporter. PPT, phosphoenolpyruvate/phosphate translocator. PPDK, pyruvate phosphate dikinase. Supplementary Figure S2: qRT-PCR validation of the expression levels of six key genes regulating lipid metabolism at the S3 stage. Note: The single-gene transcript validated was the transcript with the highest FPKM value among all transcripts annotated for that gene in the transcriptomic analysis. Different letters above the bars indicate significant differences (*p* < 0.05) in the relative expression of each gene among different cultivars under the LSD test. Supplementary Table S1: Method-validation parameters for GC-FAME analysis of fatty acids, including limit of detection (LOD), limit of quantification (LOQ), intra-day and inter-day precision (RSD), spike recovery, linear range, and calibration standard points. Supplementary Table S2: Metabolomics data. Supplementary Table S3: Transcriptomics data. Supplementary Table S4: Annotated TG metabolic pathway unigenes. Supplementary Table S5: Lipidomics data. Supplementary Table S6: Differences of the identified 216 DAMs in three comparison groups. Supplementary Table S7: Annotated Starch-Sucrose metabolic pathway unigenes.
